# Real-Time Underwater Image Recognition with FPGA Embedded System for Convolutional Neural Network

**DOI:** 10.3390/s19020350

**Published:** 2019-01-16

**Authors:** Minghao Zhao, Chengquan Hu, Fenglin Wei, Kai Wang, Chong Wang, Yu Jiang

**Affiliations:** College of Computer Science and Technology, Jilin University, Changchun 130012, China; zhaomh17@mails.jlu.edu.cn (M.Z.); hucq@jlu.edu.cn (C.H.); weifenglin@jlu.edu.cn (F.W.); wangkai87@jlu.edu.cn (K.W.); wangchonghrb@jlu.edu.cn (C.W.)

**Keywords:** CNN, FPGA, image recognition, underwater smart device

## Abstract

The underwater environment is still unknown for humans, so the high definition camera is an important tool for data acquisition at short distances underwater. Due to insufficient power, the image data collected by underwater submersible devices cannot be analyzed in real time. Based on the characteristics of Field-Programmable Gate Array (FPGA), low power consumption, strong computing capability, and high flexibility, we design an embedded FPGA image recognition system on Convolutional Neural Network (CNN). By using two technologies of FPGA, parallelism and pipeline, the parallelization of multi-depth convolution operations is realized. In the experimental phase, we collect and segment the images from underwater video recorded by the submersible. Next, we join the tags with the images to build the training set. The test results show that the proposed FPGA system achieves the same accuracy as the workstation, and we get a frame rate at 25 FPS with the resolution of 1920 × 1080. This meets our needs for underwater identification tasks.

## 1. Introduction

The underwater environment is very complex. We usually use sensors, such as sonar and cameras, to collect underwater object information [1,2,3]. The recognition degree of cameras is higher than that of sonar, but higher computational complexity is required in analyzing the whole data. The autonomous intelligent underwater vehicle (AUV) is an underwater robot, which has the advantages of a large range of activities, safety, and intelligence, and has become an important tool for the completion of various underwater tasks. It can be used for laying pipelines, undersea inspection, data collection, drilling support, submarine construction, maintenance, and repair of underwater equipment, etc. AUV was unable to communicate with staff because electromagnetic waves could not communicate over long distances in the water. Therefore, the AUV seldom processes or analyzes the images captured by the camera in real time, which usually stores sampled images on the built-in memory chip [4]. Moreover, AUV cannot respond to the surrounding environment in time [5]. When the underwater vehicle travels to dangerous areas, it can’t make timely adaptations, such as evasion or bypass, which are easy to cause damage to the hull. Regarding the traditional solution, the sonar of large AUVs is used to detect objects. However, sonar detectors must be kept a few meters or more away from the objects. Since small AUVs are navigating in shallow water, the physical damage to the AUVs cannot be prevented, and the AUV’s working area is limited. To solve this problem, we plan to implant chips into the underwater vehicle to improve the capability of immediate image processing. In this way, the AUV can respond to the image information provided by the camera, make evasive behaviors to dangerous objects, track marine life, and surround and photograph environmental resources. However, the computing capability of traditional low power CPU is very weak, and it must be paired with a GPU for these applications [6]. The navigation system mainly occupies the power supply of the submarine, whose power cannot provide any more than 100 watts to the traditional microcomputer [4]. For those reasons, we propose the FPGA chip with low power consumption to perform the real-time image recognition. Image recognition, in the context of machine vision, is the ability of software to identify objects, places, people, writing, and actions in images [7]. With the processing of FPGA, AUV is able to automatically distinguish between driving areas like seawater, and non-driving areas, such as rocks and algae. When rocks are found, AUV takes steps to avoid collisions. When confronted with algae, AUV needs to prevent the propeller from being caught. Therefore, AUV can respond in a timely manner to different environments.

FPGAs have three advantages: (1) High-speed communication interface [8,9,10]. FPGA can be used for high-speed signal processing, especially for high sampling rate from sensors. The FPGA can filter the data and reduce the data rate, which makes the complex signal easy to process, transmit, and store. (2) Fast digital signal processing. FPGA is suitable for high-real-time data processing, such as images [9,10,11], radar signals, and medical signals, more efficiently than CPU [12]. (3) Higher level of parallelism [7]. FPGA has two characteristics of parallelism and pipeline [13]. Parallelism can repeatedly allocate and compute resources so that multiple modules can be computed independently, at the same time. The pipeline makes hardware resources reusable. Those two factors combined can significantly improve parallel performance [14].

Convolutional Neural network (CNN) is a mature, fast image classification algorithm [15]. In the 1960s, Hubel and Wiesel proposed the first convolutional neural network. Now, CNN has become one of the research hot spots in many fields of science, especially in the field of pattern recognition. Without complicated pre-processing of the image, it can analyze the original image directly [16]. This algorithm can make multiple data processing units paralleled, which is suitable for the parallelization of FPGA. To solve the problem of image processing in time, we design and implement an FPGA data processing system based on lightweight CNN. We expect the FPGA system has a speed of 1920 × 1080 10 fps to meet the minimum requirements for real-time image processing, while consuming no more than 10 watts. Comparing the well-trained model written in the FPGA with a workstation platform, our system is able to accurately classify the captured images in a short time with low power consumption.

The structure of the paper is as follows. In the second section, the concept of CNN and related theories are introduced. In the third section, the overall architecture of a lightweight FPGA system is described. In the fourth section, the system implementation is presented, including parallelism design and pipeline design. In the fifth section, the neural network is written into the FPGA system, and it is compared with the results of the workstation platform and embedded platform in the same video test set. In the sixth section, some related work about this paper is demonstrated. The last section shows the corresponding conclusion and future prospects.

## 2. Methodology

With the wide application of CNN, it has been achieving excellent results in many fields in recent years. The accuracy rate of simultaneous shorthand product based on CNN exceeds 95 percent, more than the level of human shorthand [15]. In 2012, the neural network method made a significant breakthrough in the large-scale image data set ImageNet, and the accuracy rate reached 84.7% [16,17,18,19,20]. In LFW face recognition evaluation database, the face recognition method based on deep neural network DeepID, in 2014 and 2015, it reached an accuracy rate of 99.15% and 99.53%, respectively. This is even higher than the accuracy of humans, at 97.53% [20,21]. In the smart games area, in 2016, AlphaGo, of Google’s DeepMind division, bet Go world champion Lee Sedol, ranked 9-dan professional [22]. On 18 October 2017, the DeepMind team released the strongest version of AlphaGo, code named AlphaGo Zero. However, CNN’s calculation is enormous, and the power consumption of computing equipment has been tremendous in recent years. Therefore, researchers focus on the low-power FPGA chip to implement the algorithm [23,24,25,26,27].

In 2016, Wang et al. designed the deep learning accelerator unit (DLAU), which is a scalable accelerator architecture for large-scale deep learning networks, using field-programmable gate array (FPGA) as the hardware prototype [23]. The same year, Qiu et al. presented an analysis of a state-of-the-art CNN model and showed that Convolutional layers are computational centric and Fully-Connected layers are memory-centric. Next, the dynamic-precision data quantization method and a convolver design that is efficient for all layer types in CNN is proposed to improve the bandwidth and resource utilization, and a prototype of FPGA system is designed and implemented [24]. In 2017, Guo et al. used optimization approach to find the optimal allocation plan for Digital Signal Processing (DSP) resources. On a Xilinx Virtex-7 FPGA, their design approach achieved performance over the state-of-the-art FPGA-based CNN accelerators from 5.48× to 7.25×, and by 6.21× on average, when they evaluated the popular CNNs [25]. We will refer to their prototype to optimize the FPGA system architecture, and improve the overall performance of the system by using pipeline and parallel technology.

**Figure 1:**
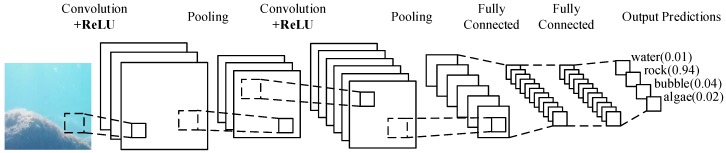
A CNN model for image recognition.

CNN is a variant of feedforward neural networks [15,28]. CNN uses convolution to reduce the proportion of images, while extracting different texture features in different ways, and then calculates the neural network. Convolution can not only reduce the computational amount of neural network image computation, but also extract features effectively. Thus, CNN includes input, several convolutions, pooling, neural network, and output. We usually describe the various parts of CNN as layers, so the structure of CNN includes the input layer, convolutional layer, pooling layer, full connected layer, and output layer. The outside of the input layer and the output layer is usually invisible, and we put them together and call them hidden layers. The main process of CNN is shown in Figure 1.

You can see the stone on the left of Figure 1. That is the input layer, which is understood as a several input matrix by the computer. Next is the convolutional layer. The depth of the first convolution is 3, and the depth of the second convolution is 6. The depth of the convolution determines how many texture features it can extract. Convolution can’t always get the value we want, so we usually need to insert activation function after convolution to make the appropriate adjustments. The part of the activation function is also called the activation layer, and the activation function selected in Figure 1 is ReLU. ReLU is an activation function that filters negative values. Behind the convolutional layer is the pooling layer. The combination of convolutional layer and pooling layer can appear in the hidden layer many times, which appears two times in Figure 1. In fact, the frequency of the convolutional layer and pooling layer is based on the needs of the model. Of course we also have the flexibility to use a combination of convolutional layer and convolutional layer, or a combination of convolutional layer, convolutional layer, and pooling layer. These are not limited for building the model. The most common CNN is the combination of several convolutional layers and pooling layers, like the structure in Figure 1. The full connected layer is behind a number of convolutional layers and pooling layers. The full connected layer is actually the Deep Neural Networks(DNN) structure. The output layer classifies image by Softmax activation function. To facilitate computer processing, we usually combine images of different depths into one nx1 array before DNN, which we call Flatten.

**Figure 2:**
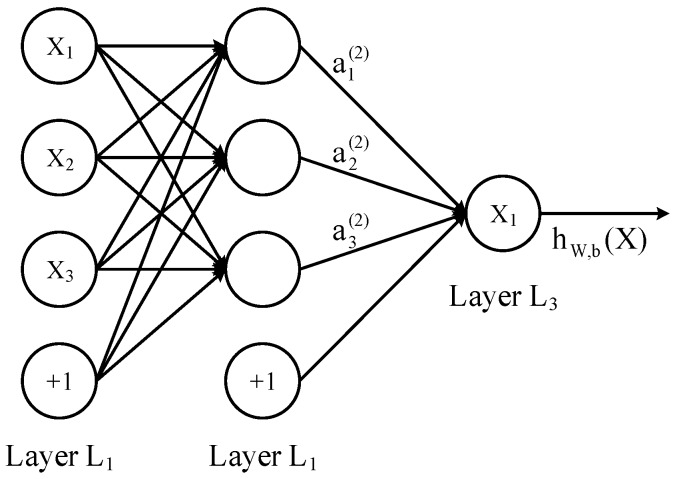
This is a simple model for DNN structure.

One DNN layer is fully connected to another layer, that is to say, one neuron in the layer *i* must be connected to any neuron of the layer *i* + 1. From a small local model, it is a combination of $z=\sum w_{i}x_{i}+b$ and an activation function σ(z). First, we define the linear relationship coefficient w. Taking the three layer DNN in Figure 2 as an example, the linear coefficients from the 4th neuron of the second layer to the 2nd neuron of the third layer are defined as $w_{24}^{3}$.

Bias *b* definition: Taking this three-layer DNN in Figure 2 as an example, the second layer of the third neuron corresponding bias is defined as $b_{3}^{2}$. Assuming that there are m neurons on the layer *l* − 1. Then for the output of the neuron *j* of the layer *l*, we get:

(1) $$a_{j}^{l}=\sigma (z_{j}^{l})=\sigma (\overset{m}{\underset{k=1}{\sum }}w_{jk}^{l}a_{k}^{l\;-\;1}+b_{j}^{l}).$$

Our goal is to use FPGA’s high-width feature to achieve a closed link between the FPGA neural network layers, while using high throughput rate to achieve fast operation based on matrix and matrix.

## 3. System Structure

In this section we will describe the structure of the FPGA system in detail and how we improve the throughput of the system through parallelism and pipeline. In the convolutional layer, we parallelize the convolution unit and use the pipeline scheme to calculate the inner product; in the full connected layer, we discuss two parallel pipeline scenarios. Taking into consideration the actual calculation of the full connected layer, we choose the second solution to eliminate the waiting time of multiplication and addition. The two points above effectively accelerate the heavy matrix operation of CNN and improve the overall efficiency of the FPGA-based CNN algorithm.

### 3.1. Architecture

**Figure 3:**
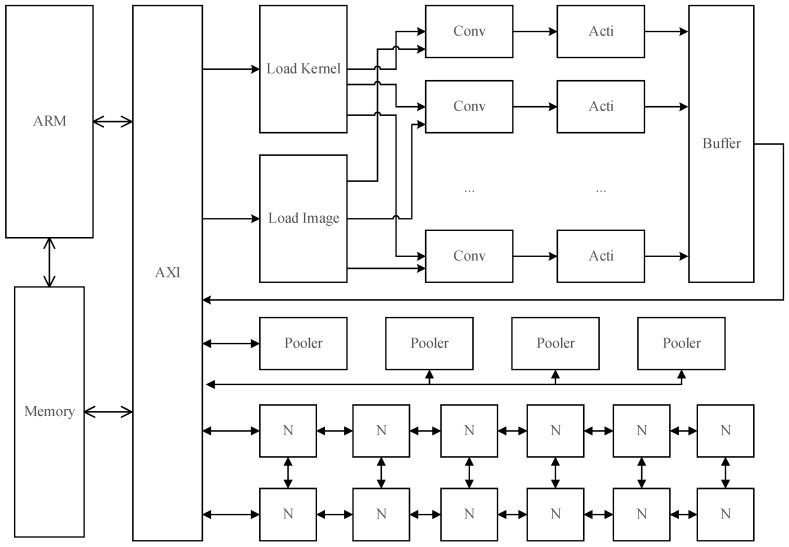
FPGA system architecture.

CNN is equipped with the gift of image recognition, while FPGA is characterized by high-speed, high concurrent performance, and low power consumption. We implement the lightweight convolutional neural network algorithm on the FPGA. We use the Xilinx ZYNQ 7000 series FPGA development board [29]. The board contains high performance ARM CPU, providing a Linux operating system for data storage, logic processing, and logging. CNN mainly includes input layer, hidden layer, and output layers. As the input layer is implemented on the ARM side, the hidden layer and output layers will be implemented on the FPGA side. The hidden layer consists of 8 convolutional units, 8 activators, 8 poolers, and 256 neural modules. The convolutional unit refers to the convolution layer of CNN. The convolutional unit corresponds to the convolution layer, with the activator in CNN corresponding to the activator layer, pooler corresponding to the pooling layer, and full connected layer is composed of the 256 neural modules. Output layers consist of 10 neural modules and interrupt modules. The structure of the system is shown in Figure 3.

In Figure 3, ARM is an onboard arm CPU. AXI bus provided by ZYNQ is a data structure between the FPGA and the memory, because the logical unit in the FPGA cannot exchange data directly with the memory without AXI bus. In Figure 3, Conv stands for convolutional unit, acti stands for activator, pooler stands for pooler module, and N stands for represented neural module. The neural module has 256 neurons that are directly connected to the AXI bus, and the neuron operation is dispatched through a neural selection register with a bit width of 16.

Considering the low power operation characteristics of FPGA, FP16 is a better choice for float format. FP16 is faster in multiplication than FP32, which gives FPGA a higher throughput rate. On the AXI bus, we have designed a total of 4 DDR controllers with 32-bit width that can provide 128 data bit width. The 128 data bit width can read 8 float numbers, 16-bit at a time. In the convolution phase, a fast convolution process for images with depth of 8 layers can be performed. In the full connected layer, the multiplication of 8 neurons can be calculated at the same time, which greatly accelerates the neural network.

### 3.2. Convolution Layer Design

In the convolutional layer, we can allow 8 convolution kernels to read the same values and calculate simultaneously for the reason that the input sources of the same depth in different convolution kernels are the same. The process of convolution [30] is shown in Formula (2):

(2) $$s_{l}(i,j)=b+\sum_{d}\left(X*W)(i,j\right)=b+\sum_{d}\underset{m}{\sum }\underset{n}{\sum }x(i+m,j+n)w(m,n)\left(1\leq l\leq L\right).$$

In Formula (7), *s* denotes the result of each point after the convolution, *l* denotes the label of the convolution, that is, the number of convolution kernel, *L* denotes the convolution depth of the current layer; the *i* and *j* denote the horizontal and ordinate, respectively. *X* denotes the image matrix and *W* represents the weight matrix required by the calculation unit, while *d* denotes the depth of the convolution, whose maximum value is the maximum depth (the number of convolution kernels) of the previous layer; *m*, *n* denotes the horizontal ordinate of the convolution kernel, and *b* refers to the bias, usually a constant obtained during training. The operation in the above formula is implemented in the convolutional unit, and in Figure 3 the 8 convolutional units can be running with a maximum depth of 8 in parallel.

Considering that the calculation in different convolution depths is independent, the result of the previous point has no effect on the result of the latter point, so we separate the calculation in different convolution depths to improve the parallel efficiency. This can reduce the computational difficulty in the formula above and cause Formula (3).

(3) $$s_{l,d}(i,j)=(X*W)(i,j)=\underset{m}{\sum }\underset{n}{\sum }x(i+m,j+n)w(m,n).$$

The parameter description of formula is the same as the formula $s_{l}(i,j)$, and finally the addition result of all the $s_{l,d}$ is the same as $s_{l}$. For this purpose, we designed 8 convolutional kernel units in each convolutional unit.

**Figure 4:**
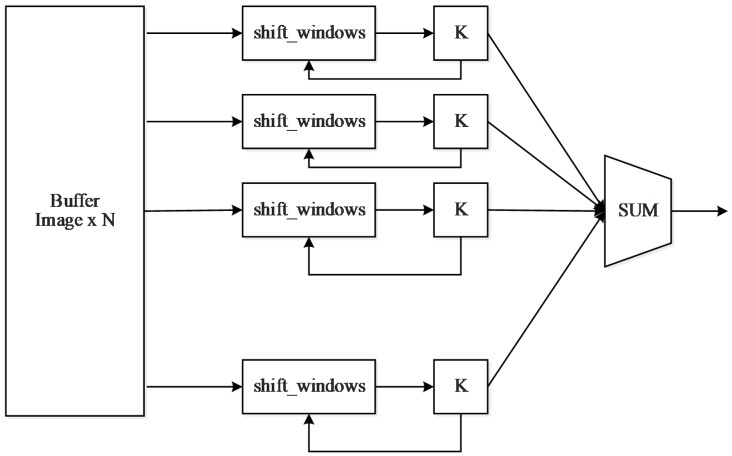
The structure of the convolutional unit.

As shown in Figure 4, the left buffer will store N images of different depths. The shift_window is a convolution shift window that holds a 3 × 3 convolution matrix. K represents the convolution unit, and the weight of the convolution matrix is stored in K. After receiving the convolution data from the shift_window, the inner product operation starts immediately. At the end of the inner convolution operation, K sends an end request to the shift_window module, and SUM will get the result at this point, which accumulates the values of all depths and bias. The result is cached in the buffer, and as the memory read request ends, the cache writes the new point back into memory.

**Figure 5:**
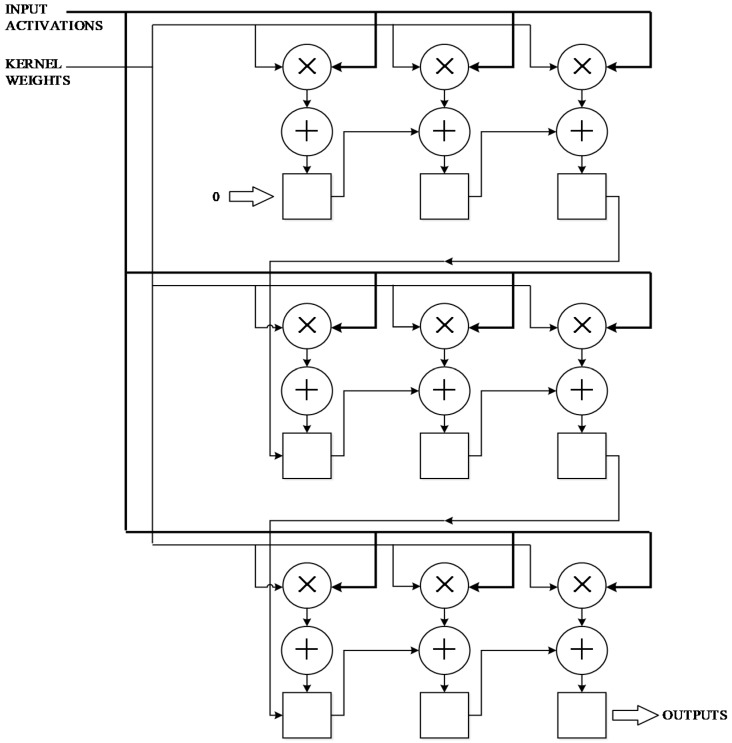
The structure of the inner product unit of Convolution Layers.

The maximum inner product of the convolution is 3 × 3, with 9 multipliers and 9 adders, as shown in Figure 5.

Since the calculation time of the multiplier and adder is greater than data loading, and the multiplier calculation time is greater than the adder. We accelerate the convolution speed by pipeline strategy.

**Figure 6:**
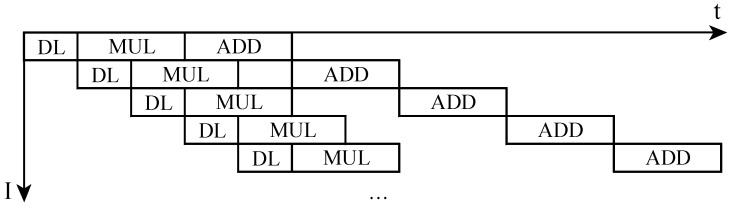
The pipeline of inner product unit of Convolution Layers.

As shown in Figure 6, each unit has three steps—loading data, multiplication, and addition. Once the data is loaded, the multiplication of each unit starts immediately. After that the data bus is vacated, the next unit loads data at once. The multiplication starts, and the adder needs to wait until the last addition finishes. Therefore it takes 9 units of adder time plus a multiplier time and a loading time to calculate a convolution. Pipeline speeds up the convolution.

Given the limited computing resources of the FPGA, a relatively simple ReLU [30] function is selected as the activation function. It’s shown in the Formula (4):

(4) $$f(x)=\{\begin{array}{c} x\\ 0 \end{array}\begin{array}{c} \left(x>0\right)\\ \left(x<0\right) \end{array}.$$

The function can effectively filter out the negative points in the image, with an easy implementation in FPGA.

**Figure 7:**
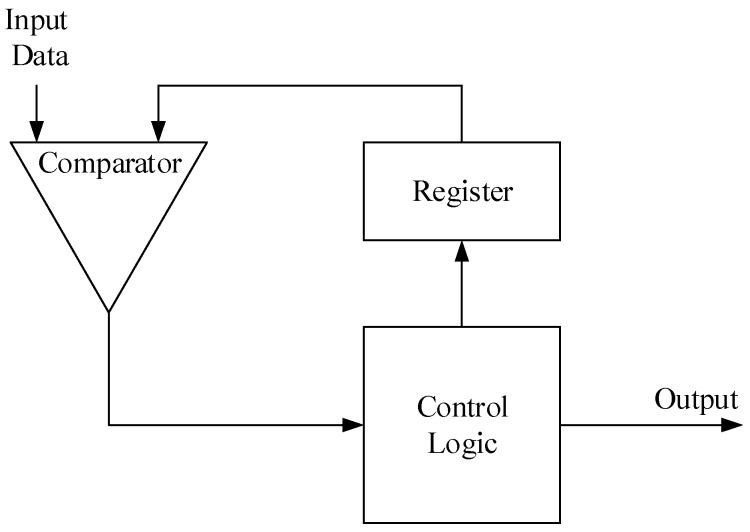
The structure of Pooler.

Pooling layer selects the Max Pooling method. The max pooling window is 2 × 2, which chooses a maximum value from 4 points as the new point; the 8 poolers can read data simultaneously from memory and read four values sequentially. The structure of the Pooler is shown in Figure 7. The register starts with a value of zero and stores the current maximum value. When one comparison finishes, Control Logic will save the result of Comparator, and Comparator will read a new point and start a new comparison. After four comparisons, Control Logic will write it back to memory.

### 3.3. Fully-Connected Layers Design

For the fully connected layer, a larger matrix operation has been generated after flattening, however our memory bus has only 128 bits and can only get eight 16-bit float numbers at the same time. Each neuron can record 16 weights. The following are two strategies to consider.

**Figure 8:**
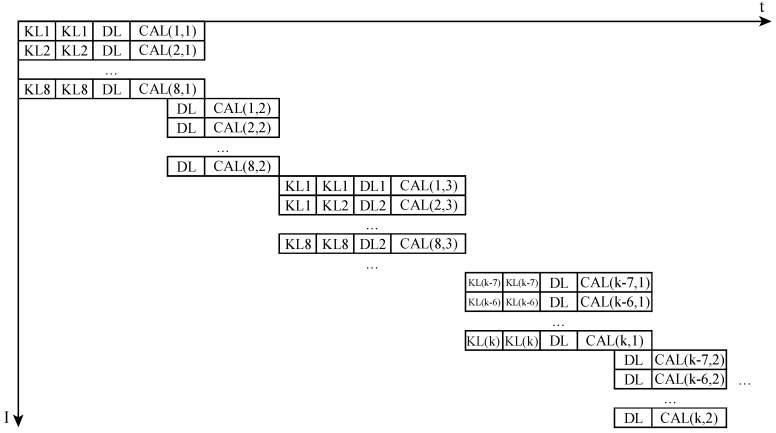
The first strategy of Full Connected layer.

Strategy 1: Load 8 × 16 weights of number 0–7 neurons, load 8 × 8 image values, and calculate. When the calculating is carried out, load 8 × 8 neurons at the same time. Calculate, and load 8 × 8 image values simultaneously. Load the following 8 × 16 weights of number 0–7 neurons, load 8 × 8 image values, and calculate. Repeat the process until all points are calculated and get the output value. Then calculate the output value of number 8–15 neuron values, and calculate the output value of numbers 16–23, until you get output of all neurons, as shown in Figure 8.

**Figure 9:**
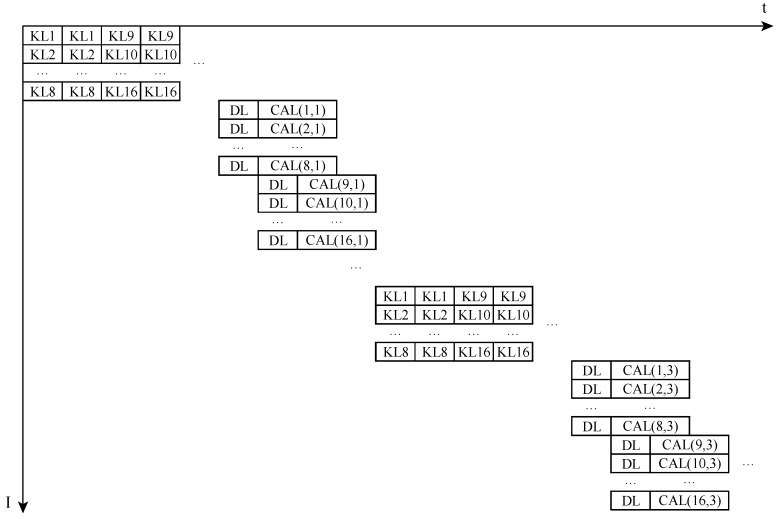
The second strategy of Full Connected layer.

Strategy 2: Load number 0–7 neuron’s 8 × 16 weights, load number 8–15 neuron’s 8 × 16 weights, and load all neuron’s weights. Then numbers 0–7 load 8 × 8 image values and calculate. When numbers 0–7 are calculating, number 8–15 loads 8 × 8 neurons at the same time, then numbers 8–15 calculate. Until all the neurons finish the work, reload all the weights of all the neurons, and then load 8 points, calculate, … until all the points are calculated. It’s shown in Figure 9.

Assuming the result of the previous layer is the matrix of *N* × 1 and *n* points on this layer, loading 8 values takes a clock cycle, and the calculation needs the b clock cycle.

For strategy 1, loading weights costs

(5) $$t_{1k}=\frac{N}{8}\cdot 2a\cdot \frac{n}{8}.$$

Calculation costs

(6) $$t_{1c}=\frac{n}{8}\cdot \frac{N}{8}\cdot b+\frac{a\cdot n}{8}.$$

The whole process needs

(7) $$t_{1}=t_{1k}+t_{1c}=\frac{\left(N\left(b+2a\right)+a\right)n}{64}.$$

For strategy 2, loading weights costs

(8) $$t_{2k}=\frac{N}{8}\cdot 2a\cdot \frac{n}{8}.$$

Calculation costs

(9) $$t_{2c}=\frac{\left(\left(n/8-1\right)a+b\right)\cdot N}{8}.$$

The whole process needs

(10) $$t_{2}=t_{2k}+t_{2c}=\frac{\left(8\left(n-1\right)a+8b+2a\cdot n\right)\cdot N}{64}.$$

The difference between Scenario 1 and Scenario 2 is

(11) $$\Delta t=t_{1}-t_{2}=\frac{\left(b-a\right)\left(n-8\right)N+8an}{8}.$$

It is known that calculation is slower than data loading, and we note that there are at least 2 neurons in the output layer. So n is greater than or equal to 2. When n is greater than or equal to 8, it is constant, but according to experience, only a few layers’ neuron number less than 8, which indicates that strategy two is more effective than the strategy one in most cases, so we choose strategy two as the pipeline design of full connected layer.

## 4. Experiment Setup

Based on our ideas in section three and four, we wrote FPGA logic code. In this section, we will test our logic. Our experimental data comes from the video, collected by underwater robot in the underwater world of the Tianchi Lake of Changbai Mountain, which is a 1920 × 1080, 30 FPS video. We extracted the clearest images by one frame per second, divided one picture into 480 × 360 pictures, and labeled the data source. After pre-procession, we trained the dataset on the workstation and exported the best-trained neural network model. Instantiating on the FPGA, we compared two strategies. After that, we compared the better one with the training platform and embedded platform.

### 4.1. Evaluation Criteria

Because of the complicated training process and the large amount of computation, we trained the model on the high performance workstation. The evaluation criteria of the experiment were accuracy and categorical cross entropy loss [15]. For each picture, the output is shown in the matrix. The first element of the column in the matrix corresponds to the probability of the first category, the element in the second column and the second row corresponds to the probability of the second class of classification, and the element in the n row and n column corresponds to the probability of the n category. It’s shown in Formula (12):

(12) $$result=\left[\begin{array}{ccc} 0.13 & 0 & 0\\ 0 & 0.72 & 0\\ 0 & 0 & 0.15 \end{array}\right].$$

From the results in Formula (12), the result of the second line is the highest probability, so we assume that the picture belongs to the second category. Accuracy calculates the rate of the correct number in all picture categories.

(13) $$Accuracy=\frac{n_{pt}}{n}.$$

In the Formula (13), *n* is the actual number of samples in a category, and the *n_pt_* is the correct number of the result in the test.

When calculating multi-classification, the difference of categorical cross entropy loss [30] exists between the predicted data and the true data. The lower the value is, the better the model we get. The formula is as follows:

(14) $$loss=-\sum_{i=1}^{n}{\overset{\wedge }{y}}_{i1}\log y_{i1}+{\overset{\wedge }{y}}_{i2}\log y_{i2}+\ldots +{\overset{\wedge }{y}}_{im}\log y_{im}.$$

In Formula (14), *n* is the number of samples, and *m* is the number of categories; $\overset{\wedge }{y}$ is the probability that the sample is expected to be. If the tag is marked as a certain class, the expected probability is 1, otherwise it is 0. Further, *y* is the probability of the test result, as shown in results above. Even though the accuracy rate is 100 and all samples have a predictive probability of 0.6, for 100 samples, the loss is 22.18. So we expect the loss of the model to be as low as possible, and the loss values to be as similar as possible in the test set and in the predicted set.

### 4.2. Experiment Result

We received three effective videos, with 20 min effective duration. From 20 min of video, we got 1200 pictures. The videos come from three areas. The first area is about the images that the submersible collects when it just enters into the water, so we mark it as shallow water. The second area is about the images that the submersible collects when it dives to a certain depth and more rocks and algae can be seen, so we mark it as middle water area. The third area is that the submersible reached 300 m, where the light is weak, so we mark it as a weak light area. The captured 1200 images contain 338 shallow waters, 403 middle waters, and 459 low light photos. Through dividing the images by 4:3, we get 14,400 training samples. We divide the images into four categories: water, algae, bubbles, and rocks, and we attach the label to each picture. Tianchi Lake of Changbai Mountain is a volcanic lake with no primitive fish habitats. In order to reduce the damage and cost of AUV, Tianchi Lake is selected as an experimental site for acquiring data, which can avoid the interference of fish on the submersible. In future experiments, other underwater environments and oceans would be considered. This is the first AUV travel in the Tianchi Lake, and in the future, we will consider the possible categories. The data set results are shown in Table 1.

Note that in weak light areas, the shooting ability of the camera is weak, so it is unable to obtain a valid bubble picture. And for some blurry pictures which humans cannot identify accurately, we labeled as rock, since rocks and unknown areas are dangerous. As three data sets are different, we trained each data set separately. We sampled 70 percent of the data set randomly as a training sample and 30 percent as a test sample.

The FPGA test platform used in this article was Xilinx ZYNQ 7035. The platform contains 275K logic cells, 900 DSP units, and a dual-core arm, which meets our demand for the number of logical units and float computing capacity. The platform can run Linux, which is easy to store data and debug.

The training experiment platform selected in this paper: Hardware platform: E5 1230v4 64GDDR4 1080Ti SLI, software platform: Ubuntu 18.04, Python 3.6, tensorflow 1.9.1 and Keras2.2.4.

The embedded experiment platform selected in this paper: Hardware platform: Raspberry Pi 3B, software platform: Raspbian, Swap 2GB, Python 3.6, tensorflow 1.9.1 and Keras2.2.4

The power consumption of the Raspberry Pi is less than 10 watts, and it can work as a personal computer. It is suitable for computing with low power consumption.

In order to correspond to the structure of our FPGA design, we use two 3 × 3 convolution kernels, and take three convolutions. The depth of convolution is 8, 4, 4, and the step is 1. There are three layers in the full connection layer. The epochs are 10, the activation function is ReLU, and dropout is 0.5. The loss function used in training is cross-entropy cost function, and the optimal result of the training model is as follows.

From Table 2, the accuracy of the training model in the training set is more than 0.85. As the underwater submersible moves at speed, the camera always captures blurred images, which human eyes cannot identify accurately. We export the Keras model weights as h5 and adjust them to the parallel-loaded file format and write these into the FPGA Flash. The results of the two strategies are shown in Table 3.

In Table 3, loss_1_, acc_1_, and time_1_ are loss, accuracy, and computing time of the first strategy, respectively, and loss_2_, acc_2_, and time_2_ are results of the second strategy, respectively. They show the same loss and acc. The second strategy takes half the time of the first one. So in our model, the second strategy is a better choice.

The experimental results of the FPGA platform with the second strategy and contrast platform are shown in Table 3.

In Table 4, Test1, Test2, and Test3, respectively, represent shallow water, middle water, and weak light layers. Loss_f_, acc_f_, and time_f_ are loss, accuracy, and computing time of the FPGA, respectively; loss_c_, acc_c_, and time_c_ are the loss, accuracy, and computing time of the comparison training experiment platform, and loss_e_, acc_e_, and time_e_ are the results of embedded platform. The experiment shows that the accuracy of the FPGA structure is the same as the comparison platform. Loss is slightly higher than the workstation platform, because we use the non-standard 16-bit float multiplication and addition. The speed non-standard 16-bit float multiplication and addition performance is high, compared with the real value, which will lead to a certain error. The error only affects the loss value, and will not have too much impact on the accuracy rate. From the test time, the differences between our FPGA test platform and 1080 Ti are remarkable. The workstation platform needs 200 watts on power supply, a huge power consumption that underwater submersibles cannot provide. Our FPGA platform’s average power consumption is just about 9.5 watts. The three test sets contain 1235, 1451, and 1652 pictures, respectively. Therefore, the average processing speeds of the FPGA are 309, 353, and 367 images per second. The lowest speed is 309 480 × 360 images per second, which is equivalent to 25 1920 × 1080 images, which is 1920 × 1080 25 fps. The processing speed reduced by 50 percent and power consumption reduced by 30 percent, and the processing power requirements for 1920 × 1080 10 fps of underwater cameras can almost be met. Although the Raspberry Pi can achieve the same loss and accuracy, the processing time of the Raspberry Pi does not meet our needs.

## 5. Conclusions

Based on the parallelism and pipeline technology of FPGA, we designed an underwater image recognition system with CNN and instantiate it on an FPGA platform. We exported the trained model from the workstation platform and inputted it into the FPGA system. In the test phase, compared with the workstation, the final test result is the same as the train workstation. The system needs low power consumption, and its computing capability is satisfied with the requirement of the underwater camera, which is 1920 × 1080 10 fps, possessing the highest performance of 1920 × 1080 25 fps. Our system can analyze the image collected by the camera of AUV in time, which enhances its perception of the surroundings, and allows it to take timely action in dangerous environments. For the following work, we will improve the overall performance of our system. The convolution operation will be expanded to the scale of more than eight convolution kernels, while non-standard size images and convolution operations with depths less than four will be optimized.

## Figures and Tables

**Table 1:** Dataset.

	Sum	Water	Algae	Rock	Bubble
shallow water	4056	1661	652	1188	555
middle water	4836	1789	877	1820	350
weak light	5508	1456	1879	2173	-

**Table 2:** Training optimal parameters.

	Loss	Accuracy
Shallow water	0.59	0.887
Middle water	0.44	0.923
Weak light	0.43	0.861

**Table 3:** Comparison of two strategies.

	Loss_1_	Acc_1_	Time_1_	Loss_2_	Acc_2_	Time_2_
Shallow water	0.61	0.872	8.0 s	0.61	0.872	4.0 s
Middle water	0.48	0.920	8.0 s	0.48	0.920	4.1 s
Weak light	0.44	0.865	9.9 s	0.42	0.865	4.5 s

**Table 4:** Comparison of results.

	Loss_f_	Acc_f_	Time_f_	Loss_c_	Acc_c_	Time_c_	Loss_e_	Acc_e_	Time_e_
Test1	0.61	0.872	4.0 s	0.60	0.872	<1 s	0.60	0.872	479.6 s
Test2	0.48	0.920	4.1 s	0.48	0.920	<1 s	0.48	0.920	572.9 s
Test3	0.44	0.865	4.5 s	0.42	0.865	<1 s	0.42	0.865	656.3 s
